# Cortisol level dysregulation and its prevalence—Is it nature's alarm clock?

**DOI:** 10.14814/phy2.14644

**Published:** 2020-12-19

**Authors:** Carol Jones, Christopher Gwenin

**Affiliations:** ^1^ School of Natural Sciences Bangor University Bangor UK; ^2^ Department of Chemistry Xi'an Jiaotong‐Liverpool University Suzhou Jiangsu Province P.R. China

**Keywords:** cortisol, hormone, HPA axis, inflammation, noncommunicable disease

## Abstract

This review examines the stress hormone cortisol which plays an important role in regulating and supporting different bodily functions. Disruption in cortisol production has an impact on health and this review looks at a wide range of papers where cortisol has been indicated as a factor in numerous chronic conditions—especially those which are classed as “noncommunicable diseases” (NCDs). Timely detection, screening, and treatment for NCDs are vital to address the growing problem of NCDs worldwide—this would have health and socioeconomic benefits. Interestingly, many of the papers highlight the pro‐inflammatory consequences of cortisol dysregulation and its deleterious effects on the body. This is particularly relevant given the recent findings concerning COVID‐19 where pro‐inflammatory cytokines have been implicated in severe inflammation.

## Graphical abstract

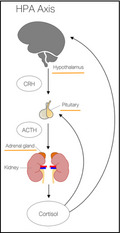

## 1 INTRODUCTION

**Figure 1:**
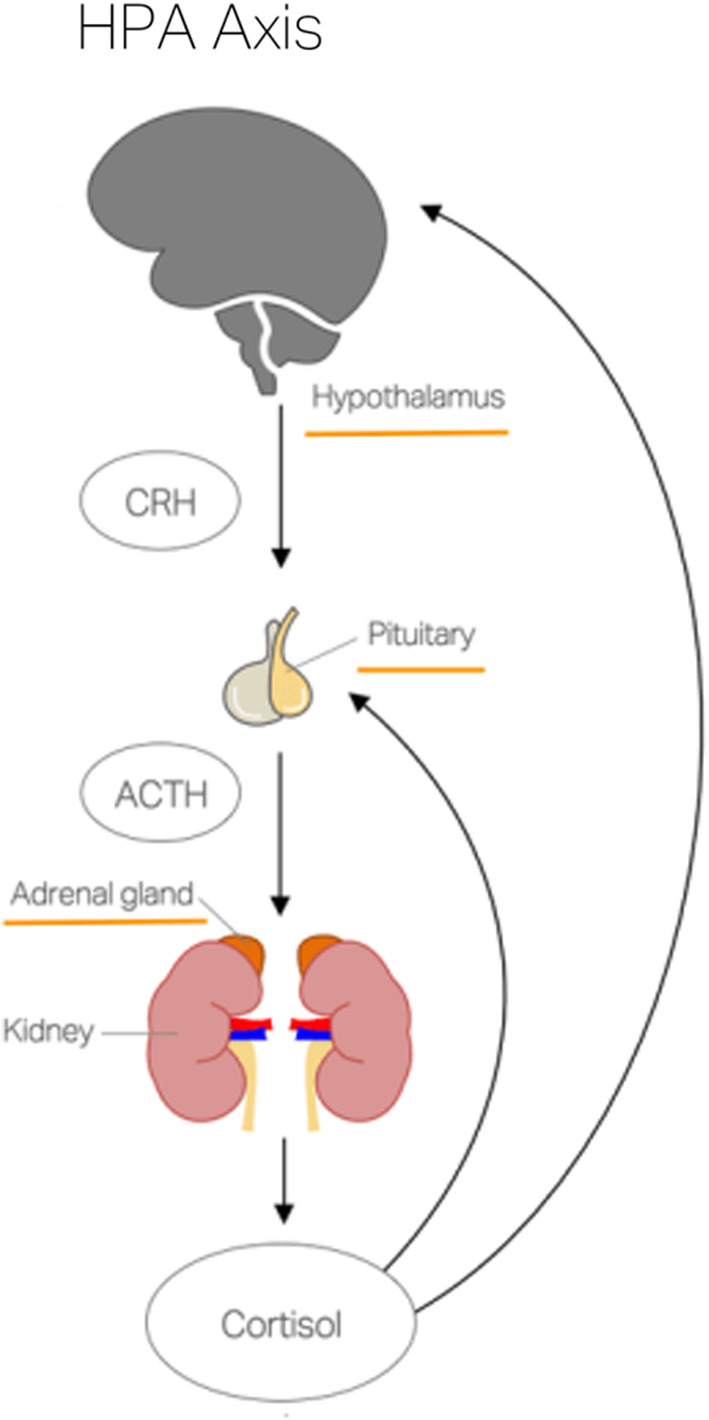
An overview of the HPA axis

Cortisol is a steroid hormone which plays a major part in the body's metabolic reaction to stress—be it physiological: illness, injury, and trauma; or psychological: mental ill‐health. Its more common effect is often described as the “flight or fight” response, which allows the body to react quickly to a perceived “threat.” Cortisol is secreted by the adrenal glands and it is a product of the complex interaction between the hypothalamus and pituitary glands in the brain and the adrenal glands, which are located at the top of each kidney—known as the hypothalamic‐pituitary‐adrenal (HPA) axis (HPA axis, see Figure 1; DeMorrow, 2018; Sapolsky et al., 2000). Cortisol is released for many hours after encountering a stressor and once the required concentration of cortisol is achieved, the cortisol exerts negative feedback to the hypothalamus, returning systemic homeostasis (Kyrou et al., 2006; Walker, 2006).

The majority of bodily cells have cortisol receptors; therefore, it plays an important role in regulating and supporting the different functions in the body: cardiovascular, metabolic, homeostatic, cellular health, and the central nervous system and it also supports the developing fetus in pregnancy. For instance, glucocorticoid receptors are manifest in the majority of bodily cells, and their function is anti‐inflammatory and immunosuppressive. Mineralocorticoid receptors are produced in the adrenal cortex and are responsible for electrolyte and fluid balance. Hence, cortisol is a vital hormone that protects health and well‐being having the qualities of being immunosuppressive and anti‐inflammatory (Bellavance & Rivest, 2014; Wamil & Seckl, 2007).

Cushing's syndrome (hypercortisolism) is caused by high levels of circulating cortisol (glucocorticoid), with individuals presenting with weight gain in the face, abdomen, and chest; it also causes skin changes, mood swings, osteoporosis, and high blood pressure (Kyrou et al., 2006; NHS; The Pituitary Foundation, 2018). The glucocorticoid excess typical of Cushing's syndrome can also lead to the development of diabetes mellitus (Barbot et al., 2018) Diabetes mellitus falls into the category of metabolic diseases (MDs) which will be discussed later in this review. In contrast, Addison's disease (primary adrenal insufficiency) is characterized by low cortisol levels. It is a serious autoimmune disease which causes damage to the adrenal glands. The onset of the disease is slow and therefore difficult to diagnose; symptoms include fatigue, muscle and weight loss, mood swings, and skin changes (National Institute for Health & Care Excellence, 2016; NHS).

Importantly, both high and low levels of cortisol, however, are indicated in other chronic conditions which will be discussed in this review.

**Figure 2:**
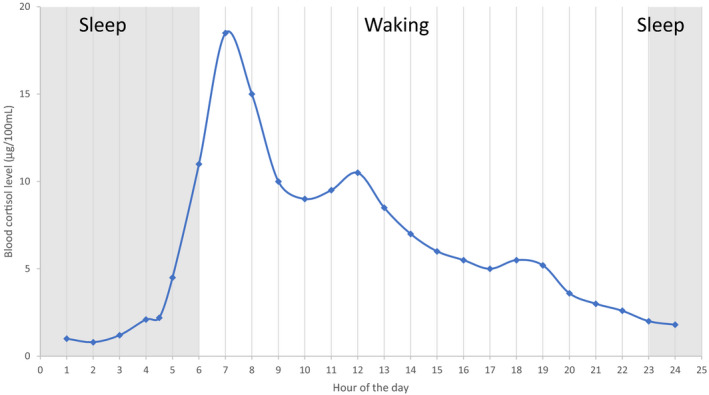
Circadian Cortisol Secretion Pattern (Lovallo &amp; Thomas, 2012)

Cortisol secretion follows a natural 24‐hr cycle (see Figure 2). In healthy individuals, peak levels are reached about 30 min after waking—this early peak is known as the cortisol awakening response (CAR). Levels decline throughout the day, with lowest levels occurring during the early sleeping phase (Clow et al., 2010; Fries et al., 2008). However, prolonged exposure to stressors can lead to the overstimulation of the HPA axis resulting in fluctuating cortisol levels. This dysregulation of the HPA axis and its resultant disruption of the 24‐hr cycle have been the subject of several studies which seek to establish a link between cortisol levels and ill‐health (Adam et al., 2017; Rao & Androulakis, 2018).

The World Health Organization (WHO) in its report “Mental health, resilience and inequalities” (Friedl, 2009) recognizes the role of low‐level prolonged psychological stress in the hyperactivation of neuroendocrine, cardiovascular, and immunological systems. It highlights the link to cortisol production and how this is implicated in the development of chronic conditions such as coronary heart disease, stroke, diabetes, high blood pressure, MDs, and mental illness. The WHO states that these diseases, in which HPA axis dysregulation is indicated, form a part of a group of illnesses termed as “noncommunicable diseases” (NCDs)—cardiovascular disease (CVD) accounts for the majority of deaths with 17.9 million people dying annually, and diabetes accounts for 1.6 million deaths. The WHO highlights the need for timely detection, screening, and treatment to address the burgeoning issue of NCDs worldwide (World Health Organisation, 2018). Consequently, this review looks at studies in which cortisol has been identified as being a significant biomarker associated with various chronic conditions.

## 2 CARDIOVASCULAR DISEASE

Cardiovascular disease refers to conditions which affect the heart or blood vessels; these include coronary heart disease, strokes, peripheral arterial disease, and aortic disease (NHS). The majority of NCDs worldwide are the result of cardiovascular disease with 17.9 million people affected annually (World Health Organisation, 2018). In this section, we look particularly at the role of cortisol in stroke and heart disease.

### 2.1 Stroke

Stroke is a life‐threatening condition that occurs when there is an interruption in the blood supply to the brain. There are two causes of stroke: ischemic (which accounts for 85% of all cases)—where a blood clot interferes with the blood supply; and hemorrhagic—where a blood vessel supplying blood to the brain ruptures (NHS).

Stroke is the second most common cause of death worldwide—there are 6.7 million deaths each year. In the UK, there are over 100,000 strokes each year and there are 1.2 million stroke survivors—two‐thirds of whom are left with a permanent disability. The cost to the NHS and social care agencies of each stroke patient is approximately £45,000 over 5 years (Stroke Association, 2017).

Stress‐induced raised cortisol levels (hypercortisolemia) lead to the risk factors implicated in stroke pathology (raised blood cholesterol, triglycerides, blood sugar, and hypertension), with prolonged long‐term stress affecting blood clotting—blood becomes stickier, thus increasing the incidence of stroke (University of Rochester Medical Center, 2020).

Several studies (Christensen et al., 2003; Iranmanesh et al., 2017; Kwon et al., 2015; Olsson et al., 1992; Zi & Shau, 2013) have focused on the negative impact of hypercortisolemia in poststroke recovery. A Chinese study (Zi & Shau, 2013) identifies cortisol as a significant prognostic marker of functional outcome and death poststroke. The study reported elevated cortisol levels in acute ischemic stroke patients—there was also a correlation between increased cortisol levels and stroke severity. The study attributes the hypercortisolemia to stress induced by the stroke. These raised cortisol levels were also consistent with neurological and cognitive deficits and poor outcomes.

Slowik *et al* (Slowick et al., 2002) in their study also recorded raised cortisol levels in stroke patients and noted an absence of cortisol circadian variation. The study also points to the correlation between increased cortisol and poor outcomes—with patients suffering from confusion, delirium, and depression. Cortisol levels were found to be raised several months poststroke. Interestingly, the authors posit that the raised serum cortisol levels, rather than being solely a stress response per se, are indicative of an inflammatory response, which is demonstrated by the presence of inflammatory markers. Supporting this Tene *et al* (Tene et al., 2018) found that subjects with high bedtime cortisol levels displayed inferior memory and functioning, this is consistent with HPA axis dysregulation.

A critical literature review (Mitchell, 1997) also highlights the fact that HPA axis dysregulation is present in up to 40% of stroke patients. It draws attention to studies which suggest a correlation between large neurological lesions and hypercortisolemia, resulting in poor outcomes and prognoses. It postulates that sustained high levels of glucocorticoids have a deleterious effect on the brain and its ability to recover; it goes on to advocate the timely use of antiglucocorticoids poststroke. Another study (Marklund et al., 2004) also supports these findings, that hypercortisolemia is indicated in poor outcomes and mortality poststroke. However, they also report an increased mortality in poststroke patients with low cortisol levels (hypocortisolemia)—they attribute this to a dysfunction in glucocorticoid secretion which in turn affects homeostasis. They also point to the incidence of hypocortisolemia in septic shock and the role of supplementary hydrocortisone therapy in such cases of adrenal insufficiency.

Barugh et al (Barugh et al., 2014) in their wide‐ranging systematic review report that the majority of studies looking at acute stroke patients found high cortisol levels in the first week poststroke; these higher levels were consistent with poststroke complications such as dependency, delirium, depression, and mortality. A loss of diurnal variation in severe strokes, leading to greater severity of illness, was also found to be significant in many studies. It was also noted that the diurnal variation was not affected in those suffering a less severe stroke. The authors caution other causes of HPA axis dysregulation, such as aging, stress, neurodegenerative disease, and post‐acute stroke brain injury. The authors conclude by highlighting the need for further research in this field.

The Stroke Association (Stroke Association, 2017) states that one‐third of stroke survivors experience depression poststroke and over 50% suffer from anxiety in the 10 years following a stroke. Several studies (Kwon et al., 2015; Olsson et al., 1992) cite dysregulation of the HPA axis as a factor in poststroke depression (PSD). In particular, a reduced CAR has been recorded in depressed poststroke patients (2 months plus poststroke) and restoration of the HPA axis function may be beneficial to PSD patients leading to the alleviation of their depressive symptoms and improved quality of life (Kwon et al., 2015).

### 2.2 Heart disease

Prolonged periods of stress and the resulting exposure to raised cortisol levels have been shown to have a significant effect on heart health—leading to an increase in blood cholesterol, triglycerides, blood sugar, blood pressure, and truncal obesity. These are all risk factors in heart disease which can lead to a build‐up of plaque deposits in the arteries (atherosclerosis); this affects blood clotting making blood stickier thus increasing the risk of cardiovascular disease (University of Rochester Medical Center, 2020). The British Heart Foundation recognizes the influence of emotional stress on the biological processes involved in heart disease; they are conducting research investigating the role of cortisol in the disease process by looking at how cortisol regulates inflammation; in particular, they are interested in the possibility that overexposure to cortisol results in cells becoming desensitized, resulting in vascular inflammation (British Heart Foundation, 2020).

Several studies (Brotman et al., 2007; Dekker et al., 2008) have linked the effects of stress with cardiovascular disease; Kumari *et al* (Kumari et al., 2011) assert that in response to psychological stress, dysregulation of the HPA axis occurs. They report flat slopes in salivary cortisol and raised evening cortisol levels as being a predictor of cardiovascular deaths in middle‐aged adults.

Dekker *et al* (Dekker et al., 2008) assert that an increase in total cortisol correlates with the incidence of carotid artery plaques as found in vascular atherosclerosis. They also refer to the increasing evidence linking cortisol levels and inflammation; like Slowik *et al* (Slowick et al., 2002), they point to the effect of glucocorticoids on the blood vessels and their role in inflammation. Ikeoka *et al* (Ikeoka et al., 2010) cite the role of adipose tissue which is linked to glucocorticoid production and its role in inflammation as a factor in cardiovascular disease. Another study also highlights the deleterious role of glucocorticoids in the development of CVD (Walker, 2007). It states that glucocorticoid excess is a risk factor in CVD—especially in the occurrence and progression of vascular atherosclerosis.

Numerous studies have linked raised cortisol levels with an increased risk of cardiac events. One study (Yamaji et al., 2009) cites the measurement of cortisol, together with brain natriuretic peptide and aldosterone as a predictor of cardiac events in patients with heart failure. It suggests that there is a link between the levels of cortisol and oxidative stress—as oxidative stress activates the cortisol‐mineralocorticoid receptor. Kelly *et al* (Kelly et al., 1998) in their study link increased cortisol levels to hypertension—a causal factor in CVD. Whitworth *et al* (Whitworth et al., 2005) while highlighting the role of glucocorticoid‐induced hypertension in Cushing's syndrome assert that hypercortisolemia may be a factor in up to 30% of cases of hypertension in the general population.

The studies cited show a clear link between stress and dysregulated cortisol levels, which is a significant factor in CVD.

## 3 METABOLIC DISEASE/TYPE 2 DIABETES MELLITUS

World Health Organization statistics reveal that in 2016, 1.6 million deaths were directly caused by diabetes and it was the seventh leading cause of death in 2016. The trajectory of incidences of diabetes is increasing—there were 108 million sufferers worldwide in 1980, this had increased markedly to 422 million by 2014 (World Health Organisation). A recent study estimates this figure to be 500 million in 2018—with the greatest growth in low‐income countries (Bradshaw‐Kaiser et al., 2008). Diabetes UK reports that in the UK alone, the number of people diagnosed with diabetes has more than doubled in the past 20 years—they predict that there will be more than 5 million cases by 2025. Over 90% of diabetes reported in the UK is type 2 diabetes mellitus (T2DM). These patients are more at risk of developing other diseases such as retinopathy, strokes, heart disease, kidney disease, and amputations. Diabetes UK estimates the cost of treating diabetes at £10 billion per annum—this figure represents 10% of the NHS annual budget (Diabetes.org.uk).

Metabolic disease and T2DM are viewed very much as “modern” diseases as they are deemed to be symptomatic of contemporary lifestyle choices—characterized by poor dietary habits and sedentary lifestyles—increasing visceral obesity (Bose et al., 2009; Parades & Ribeiro, 2013). Consequently, numerous studies have been undertaken that highlight the link between HPA axis activity and MD and T2DM (Bose et al., 2009; Joseph & Golden, 2018; Parades & Ribeiro, 2013; Rosmond, 2003). The HPA axis controls the production of glucocorticoid hormones, which originate in the adrenal cortex, and chronic glucocorticoid exposure is a factor in MD and T2DM. This reduces insulin sensitivity, which leads to a decrease in insulin secretion (Di Dalmazi et al., 2012). Obesity is characterized by visceral adipose tissue, which has high levels of glucocorticoid receptors (Musazaki et al., 2001).

Many studies reference the role of stress in HPA axis dysregulation and how this is expressed in terms of MD and T2DM (Bose et al., 2009; Di Dalmazi et al., 2012; Joseph & Golden, 2018; Parades & Ribeiro, 2013; Rosmond, 2003). Bose *et al* (Bose et al., 2009) highlight the role of stress and truncal obesity in MD and the role of HPA dysregulation in this process. They suggest that obesity is caused by the localized effect of cortisol metabolism on adipose tissue—Cushing's syndrome being a case in point. Interestingly, they also refer to studies which point to the incidence of chronic inflammation in obesity and MD (Hotamisligil, 2006; Lee et al., 1988), where pro‐inflammatory cytokines stimulate the HPA axis. They also cite other studies where cortisol has been shown to decrease cytokine production (Lederbogen et al., 2011).

**Figure 3:**
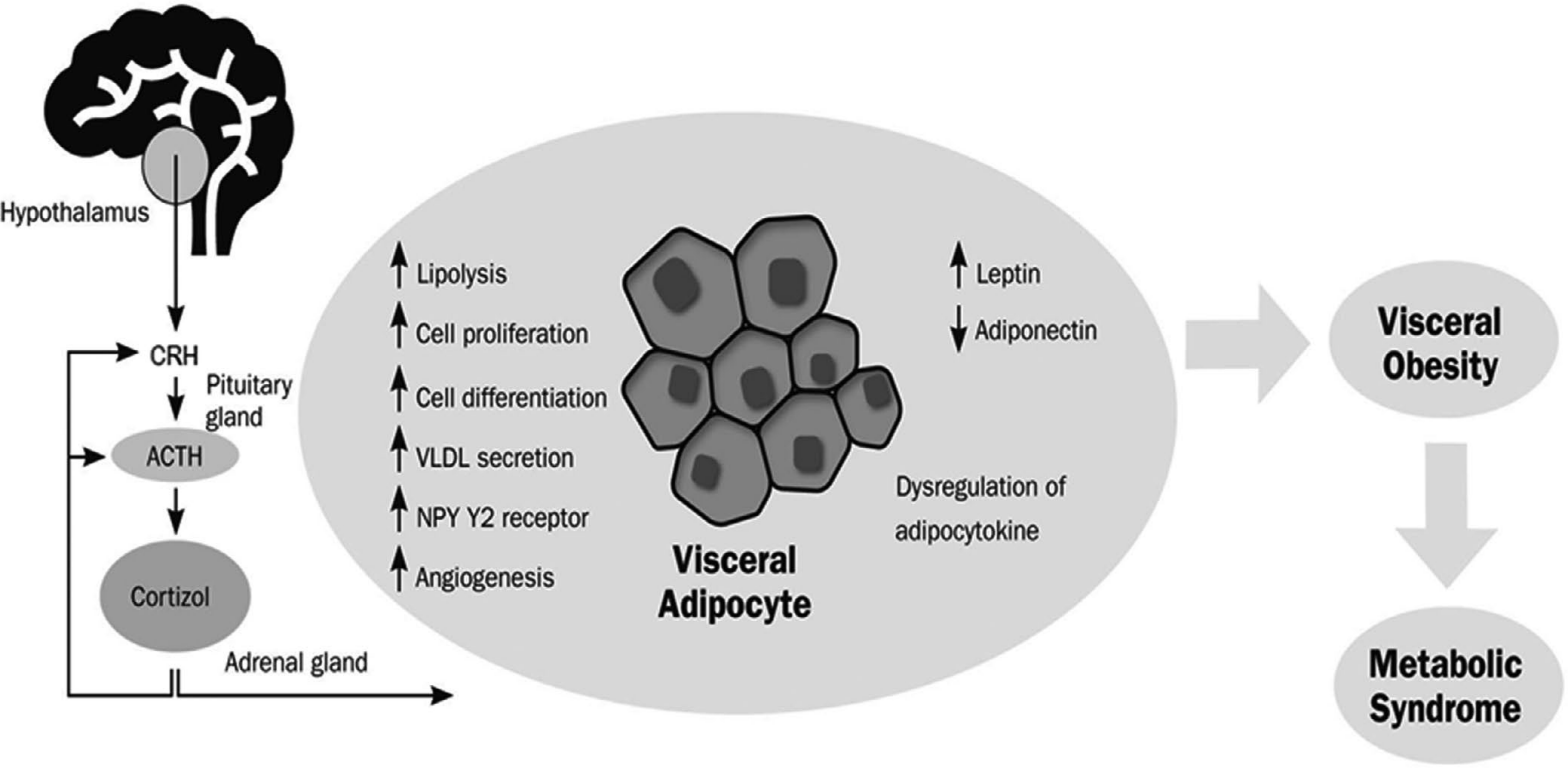
Putative mechanisms involved in GC release, visceral adipose tissue accumulation, and the pathogenesis of metabolic syndrome. Cortisol, the major glucocorticoid in humans, is secreted in response to stressful events under the control of the hypothalamic‐pituitary‐adrenal axis. Cortisol exerts several cellular and metabolic effects in adipocytes, eventually promoting visceral obesity and the development of metabolic syndrome. CRH – corticotropin‐releasing hormone; ACTH – adrenocorticotropic hormone (Parades &amp; Ribeiro, 2013)

Other studies (Di Dalmazi et al., 2012; Parades & Ribeiro, 2013) look at hypercortisolemia and metabolic dysfunction. Paredes *et al* (Parades & Ribeiro, 2013), for example, link chronic stress and HPA axis dysregulation and how this is expressed in truncal obesity and insulin resistance (see Figure 3). They also refer to the pro‐inflammatory consequences of this process—they claim that prolonged low‐grade inflammation creates a “vicious cycle” where the prolonged stress stimulates the HPA axis. Di Dalmazi *et al* (Di Dalmazi et al., 2012) advocate further research, especially in the development of anti‐inflammatory drugs to address glucose metabolism.

Several studies have investigated the role of circadian cortisol levels in T2DM patients. One study (Lederbogen et al., 2011) highlights the role of HPA dysregulation in both type 1 diabetes (T1D) and T2DM and its role in insulin resistance. They report a flattened circadian profile, with lower morning and higher evening levels. They posit that these merits further investigation to ascertain how HPA axis dysregulation is implicated in the control of glycemic levels. Similarly, Hackett *et al* (Hackett et al., 2014) also report a flattened cortisol slope in their findings. Bruehl *et al* (Bruehl et al., 2008) look particularly at the CAR and diurnal salivary profiles of T2DM patients; they also interestingly look at the link with hippocampal volume in the context of its role in HPA axis feedback regulation. They report a blunting of the CAR but a maintained diurnal pattern. They also report a reduction in hippocampal volume, which was consistent with the duration of T2DM and glycemic control.

The role of stress and the resulting HPA axis dysregulation is indicated in both MD and T2DM as characterized by an increase in visceral adipose tissue and a decrease in insulin sensitivity.

## 4 INFLAMMATION/AUTOIMMUNE

Normal glucocorticoid function plays an important role in inflammation—it works by reducing inflammation and has an immunological and metabolic effect. Prolonged exposure to high cortisol levels and the resulting HPA axis dysfunction interferes with the anti‐inflammatory and immunological processes. High circulating cortisol levels affect immune cells by binding to their receptors leading to the production of pro‐inflammatory cytokines (Glaser & Kiecolt‐Glaser, 2005; Lavretsky & Newhouse, 2012) resulting in inflammation and immune deficits and other metabolic consequences (such as MD, T2DM, Cushing's syndrome, and Addison's disease).

Inflammation is increasingly recognized as being a factor in many diseases. Numerous studies have cited the role of HPA axis dysregulation in diseases where inflammation is indicated.

Fibromyalgia syndrome (FS) is a long‐term rheumatic condition in which sufferers experience muscular and musculoskeletal pain with stiffness and localized tenderness at specific points in the body, and it also causes extreme fatigue. Studies have demonstrated a link between stress and pain—in particular, they focus on the role of cortisol levels and point to HPA dysregulation as a possible causal agent of pain (Blackburn‐Munro, 2004; Hanniabl & Bishop, 2013; McBeth et al., 2005). They posit that prolonged heightened stress leads to the blunting of the HPA axis response. In particular, the CAR was found to be reduced throughout the day (Doerr et al., 2016; Riva et al., 2010). Similarly, numerous studies have also highlighted the link between HPA dysregulation and the resulting inflammation and autoimmune response with chronic fatigue syndrome (CFS; Morris et al., 2017; Papadopoulos & Cleare, 2011; Powell et al., 2013; Silverman et al., 2010; Van den Eede et al., 2007).

The role of inflammation is also being looked at in terms of cognitive diseases. The Alzheimer's Society is studying the role of cortisol release and stress and its effects on the immune system in Alzheimer's diseases. It does caution that the picture is not a straightforward one and to this end, it is currently funding research looking at chronic stress as a risk factor in the development of AD with a view to the development of drug therapies (www.alzheimers.org.uk/research).

The COVID‐19 (Severe Acute Respiratory Syndrome Coronavirus 2) pandemic also casts light on the role of cortisol in respect of inflammation and immune deficits. The disease progression is characterized by what has been described as a “cytokine storm”—the causes of which are multifactorial; this results in extreme inflammation and a severely suppressed immune response (Hickman, 2020; Mehta et al., 2020). Isidori *et al* (Isidori et al., 2020) examine the efficacy of glucocorticoid treatment in COVID‐19 patients with adrenal insufficiency.

In June 2020, the RECOVERY (Randomized Evaluation of COVid‐19 thERapY) at the Nuffield Department of Medicine, University of Oxford established that dexamethasone, a corticosteroid used to treat inflammatory conditions, improved the survival rates in patients who required (a) ventilation—deaths were reduced by one‐third—and (b) oxygen only—deaths were reduced by one‐fifth (University of Oxford, 2020).

Extreme physiological stress and the resulting HPA axis dysregulation are significant factors in numerous conditions where chronic inflammation is an important factor.

## 5 CONCLUSION

It has been demonstrated that cortisol is a useful but somewhat complex biomarker, especially in terms of chronic ill‐health, as exemplified by the numerous conditions outlined in this review. It is important to understand its function and systemic effects and how it links to other biochemical components. The greater understanding of the complex relationship between cortisol and chronic ill‐health will enable the development of therapies.

The monitoring of cortisol levels would be advantageous in that it would facilitate timely diagnosis, monitoring, and prognosis—this would, in turn, allow for appropriate clinical planning, positive patient outcome, and allocation of health‐care resources. The World Health Organization, in particular, in relation to NCDs recognizes the importance of “early detection and timely treatment” and the positive impact of such interventions (World Health Organisation, 2018). The Royal Society for Public Health (Royal Society for Public Health, 2013) also points to the serious health and economic burden of NCDs and advocates for interventions which would lead to a reduction in the risk factors of developing such diseases—the monitoring of cortisol levels could be one such significant intervention which would address the needs of the public health agenda.

To this end, the development of a rapid, point‐of‐care diagnostic test would be beneficial—it would not require trained personnel or a laboratory and could be used in several settings: home, community, hospital, and suboptimal settings. This would facilitate more timely diagnoses and improved monitoring, thus advancing clinical outcomes and wellness. Patients could also self‐monitor their cortisol levels, giving them autonomy to manage their condition and freeing up clinicians.

## CONFLICTS OF INTEREST

The authors declare no conflict of interest.

## AUTHOR CONTRIBUTIONS

CDG and CJ conceived and designed the review. CJ wrote the manuscript. All authors read and approved the manuscript.
